# Lack of vitamin D signalling per se does not aggravate cardiac functional impairment induced by myocardial infarction in mice

**DOI:** 10.1371/journal.pone.0204803

**Published:** 2018-10-01

**Authors:** Kristopher Ford, Nejla Latic, Svetlana Slavic, Ute Zeitz, Marlies Dolezal, Oleh Andrukhov, Reinhold G. Erben, Olena Andrukhova

**Affiliations:** 1 Dept. of Biomedical Sciences, University of Veterinary Medicine Vienna, Vienna, Austria; 2 Division of Conservative Dentistry and Periodontology, School of Dentistry, Medical University of Vienna, Vienna, Austria; Max Delbruck Centrum fur Molekulare Medizin Berlin Buch, GERMANY

## Abstract

Epidemiological studies have linked vitamin D deficiency to an increased incidence of myocardial infarction and support a role for vitamin D signalling in the pathophysiology of myocardial infarction. Vitamin D deficiency results in the development of secondary hyperparathyroidism, however, the role of secondary hyperparathyroidism in the pathophysiology of myocardial infarction is not known. Here, we aimed to explore further the secondary hyperparathyroidism independent role of vitamin D signalling in the pathophysiology of myocardial infarction by inducing experimental myocardial infarction in 3-month-old, male, wild-type mice and in mice lacking a functioning vitamin D receptor. In order to prevent secondary hyperparathyroidism in vitamin D receptor mutant mice, all mice were maintained on a rescue diet enriched with calcium, phosphorus, and lactose. Surprisingly, survival rate, cardiac function as measured by echocardiography and intra-cardiac catheterisation and cardiomyocyte size were indistinguishable between normocalcaemic vitamin D receptor mutant mice and wild-type controls, 2 and 8 weeks post-myocardial infarction. In addition, the myocardial infarction-induced inflammatory response was similar in vitamin D receptor mutants and wild-type mice, as evidenced by a comparable upregulation in cardiac interleukin-1-β and tumor-necrosis-factor-α mRNA abundance and similar elevations in circulating interleukin-1-β and tumor-necrosis-factor-α. Our data suggest that the lack of vitamin D signalling in normocalcaemic vitamin D receptor mutants has no major detrimental effect on cardiac function and outcome post-myocardial infarction. Our study may have important clinical implications because it suggests that the secondary hyperparathyroidism induced by vitamin D deficiency, rather than the lack of vitamin D signalling per se, may negatively impact cardiac function post-myocardial infarction.

## Introduction

Despite extensive clinical and laboratory research on the etiology of cardiovascular diseases (CVD), they remain a primary public health concern with leading mortality rates worldwide [1, 2]. Epidemiological and animal studies have linked low serum vitamin D to hypertension, left ventricular hypertrophy (LVH), increased arterial stiffness, endothelial dysfunction, as well as, myocardial infarction (MI) incidence and pathophysiology [3–6]. However, the association between vitamin D deficiency and CVD remains a controversial issue. Recently, a large Mendelian randomisation study failed to confirm the association between vitamin D status and CVD [7].

Intervention studies have also provided conflicting evidence regarding the role of vitamin D in CVD therapy. While some studies support a cardioprotective role for vitamin D supplementation with active vitamin D analogues in both experimental MI models and in clinical studies [8–10], other studies failed to provide evidence for any beneficial therapeutic effect of vitamin D supplementation or active vitamin D analogues on vascular function and CVD outcome [11–13]. A recent review by Milazzo *et al* focusing on acute MI (AMI) and vitamin D, highlighted the necessity for well designed, adequately powered interventional trials to confirm the role of vitamin D in AMI patients [14].

It is thought that all the biological actions of the vitamin D system are mediated through the vitamin D hormone, 1α,25-dihydroxyvitamin D_3_ (1,25(OH)_2_D_3_). Vitamin D produced in the skin or taken up via the diet needs to be activated by two hydroxylation steps occurring in the liver and the kidney, respectively [15]. The cellular actions of the vitamin D hormone require the presence of the nuclear vitamin D receptor (VDR), which is found in many different cell types including cardiomyocytes, endothelial cells and macrophages [16–18]. One possible explanation for the discrepant findings in clinical and epidemiological studies is that circulating 25(OH)D levels, routinely measured to assess vitamin D status, may not reflect the 1,25(OH)_2_D_3_ concentrations in tissues.

Animal studies have provided firm evidence in favour of an important role for vitamin D signalling in the cardiovascular system. We found that vitamin D signalling regulates endothelial function by modulating the bioavailability of the vasodilator nitric oxide (NO) through the transcriptional control of endothelial-derived NO synthase (eNOS) [19]; a finding which was later confirmed in mice with endothelial cell specific VDR deletion [20]. By regulating vascular tone, vitamin D signalling could play an important role in the pathophysiology of MI and in the progression of ischemic and chronic heart failure [21]. The VDR is abundantly expressed in the heart with global VDR ablation reported to cause cardiac hypertrophy under normal resting conditions [16, 22]. More recently, studies investigating the effects of selective deletion of the VDR in cardiomyocytes support the idea that vitamin D signalling has anti-hypertrophic effects and can alter cardiomyocyte contraction and relaxation [23, 24]. Direct experimental evidence for the involvement of VDR signalling in the pathophysiology of MI is scarce. A study in global VDR knockout mice subjected to experimental MI showed that the absence of vitamin D signalling was associated with decreased survival, impaired cardiac function, elevated cardiac inflammation and fibrosis, relative to wild-type (WT) controls [8]. However, the latter study was performed on a normal mouse diet. It is well known that VDR-ablated mice on a normal diet develop severe secondary hyperparathyroidism (sHPT) due to a loss of VDR function in the small intestine, leading to a calcium absorption defect and subsequent hypocalcemia. Therefore, it is unclear whether the impairment of cardiac function post-MI in VDR-ablated mice was caused by a lack of VDR signalling in cardiomyocytes or immune cells, or by sHPT.

Growing evidence suggests an association between sHPT and CVD including hypertension, arrhythmia, structural and functional alterations in the vascular wall, diastolic dysfunction and LVH [25–31]. It has been hypothesised that the direct cellular effects of phosphate, calcium and parathyroid hormone (PTH) on endothelial cells and cardiomyocytes are involved in the development of multiple cardiovascular pathologies [32–36]. Several clinical studies have suggested an association between elevated PTH and CVD [37–41], supporting a role for increased PTH signalling in MI pathology.

Here, we sought to further explore the sHPT-independent role of vitamin D signalling in MI by inducing experimental MI in mice with a global deletion of VDR function maintained on a so-called rescue diet enriched with calcium, phosphate and lactose. The rescue diet prevents hypocalcemia and sHPT in global VDR mutants by stimulating paracellular uptake of calcium and phosphate in the gut [42, 43]. Based on the reports of an anti-hypertrophic role of VDR signalling in cardiomyocytes [22–24] and our own data linking VDR deficiency with endothelial dysfunction [19], we hypothesised that VDR deficiency would have a detrimental effect on cardiac function post-MI. However, surprisingly, we found that survival and heart function up to 8 weeks post-MI was not different between WT mice and normocalcaemic mice lacking a functional VDR.

## Materials and methods

### Animals

All animal studies were approved by the Ethical Committee of the University of Veterinary Medicine, Vienna, and by the Austrian Federal Ministry of Science and Research and were undertaken in strict accordance with prevailing guidelines for animal care and welfare (permit number BMWF-68.205/0153-WF/V/3b/2014). Three-month-old male WT and homozygous vitamin D receptor (VDR^Δ/Δ^) mutant mice carrying a functionally inactive vitamin D receptor, were bred by intercrossing heterozygous animals on a C57BL/6 genetic background [43]. All animals were housed under 12h light/dark cycles at 24°C with *ad lib* provisions of tap water and rescue diet (Ssniff, Soest, Germany) containing 2.0% calcium, 1.25% phosphorus, 20% lactose and 600 IU vitamin D/kg or a normal mouse chow containing 1.0% calcium, 0.7% phosphorus, and 1,000 IU vitamin D/kg (Ssniff).

### Acute myocardial infarction model

Acute myocardial infarction was induced in 3-month-old male mice by permanent ligation of the left descending coronary artery (LDCA). Briefly, mice were anaesthetised by intra-peritoneal injection using a ketamine/medetomidine mix (100/0.25 mg/kg) and placed under controlled ventilation with room air. Left lateral thoracotomy was performed at the 4^th^ intercostal region and the pericardium was removed to provide access to the LDCA. Ligation was placed 1–2 mm below the tip of the left atrial appendage using a 7–0 prolene suture. The pericardium was replaced and the chest and skin re-sutured. Infection was prevented and pain was managed with enrofloxacin (10 mg/kg) and buprenorphine (0.25 mg/kg) treatment, respectively. The sham operation was performed as above with the absence of coronary artery ligation. Mice were killed by exsanguination from the abdominal V. cava under general anaesthesia with ketamine/xylazine (100/6 mg/kg i.p.) either 2 or 8 weeks after surgery.

To minimize social stress animals were caged with littermates or together with female retired breeders. Daily husbandry checks were performed throughout the experiments to monitor animal health and behaviour. Weight was recorded daily for the first 5 days, then once weekly, and additionally if required for the duration of the experiment. If at any time point during the study weight loss exceeded 20% of the original weight, or if animals showed signs of severe distress such as grossly altered behaviour or reduced mobility, animals were immediately euthanized via cervical dislocation. In total 150 animals were used of which 2 mice were euthanized due to weight loss, and 24 died suddenly; cause of sudden death was mostly cardiac rupture due to thinning of the ventricular wall.

### Biochemical analysis

Urinary and serum phosphate, calcium and creatinine were measured using a Cobas c111 analyser (Roche, Mannheim, Germany). Serum PTH was detected using the mouse PTH 1–84 ELISA Kit purchased from Immutopics Inc. (San Clemente California, USA), and used according to the manufacturer’s instructions. Serum aldosterone was determined by ELISA (NovaTec Immundiagnostica, Dietzenbach, Germany) according to the manufacturer’s protocol. Absorbance was read using an Enspire 2300 multilabel reader (PerkinElmer, Massachusetts, USA). Serum concentrations of interleukin-1-beta (IL-1β) and tumor-necrosis-factor-alpha (TNF-α) were measured using the Luminex bead-based multiplex assay principle (R&D Systems Inc, Minneapolis, USA) according to the manufacturer’s instructions. Urinary NO concentrations were measured using a commercially available colorimetric assay (Cayman Chemical Company) according to the manufacturer’s protocol.

### Echocardiography

For the evaluation of cardiac function, echocardiography was performed 1 week after surgery on all animals and additionally at 7 weeks for animals involved in the 8-week study. Echocardiography was performed under isoflurane anaesthesia using a 14MHz linear-array transducer (Acuson s2000tm, Siemens). Body temperature was maintained at 37 ^o^C ± 1°C. M-mode images were captured at the level of the papillary muscles in short axis view and analysed during both systole and diastole over at least 3 consecutive cardiac cycles. Left ventricular (LV) end-diastolic diameter (LVEDD), LV end-systolic diameter (LVESD) and LV posterior and anterior wall thickness during diastole (LVPWd, LVAWd) were evaluated. As a measure of global left ventricular function fractional shortening (FS) was calculated as [(LVEDD–LVESD)/LVEDD]x100.

### Electrocardiography

Electrocardiograms (ECG) were recorded 1 week after surgery for 10 min under isoflurane anaesthesia and a constant body temperature (37 ± 1°C). A three-lead ECG was obtained by inserting electrodes into the paws. Data were transmitted to a computer via analogue to digital conversion PowerLab 15T (AD Instruments Ltd, Oxford, United Kingdom) at 4000 Hz. Data were analysed using LabchatPro software (AD instruments Ltd). Approximately 100 consecutive traces were averaged and the negative deflection of the T-wave (ST-Height) was quantified to determine T-Wave inversion.

### Central arterial pressure measurements and cardiac catheterisation

To investigate physiological cardiac adaptations arterial and cardiac pressure measurements were recorded using a SPR-671NR pressure catheter (1.4F, Millar instruments, Houston, TX, USA). Terminal procedure animals were anaesthetised using isoflurane anaesthesia with a constant body temperature (37 ± 1°C). When the paw pinch reflex had disappeared, the catheter was inserted into the ascending aorta for measurement of central arterial pressure. Cardiac pressure was measured by threading the catheter into the left ventricle down the right common carotid artery and through the aortic valve. Traces were recorded for a minimum of 2 min from both the ascending aorta and left ventricle and analysed. The mean arterial pressure (MAP), arterial pulse pressure (APP) calculated as [Systolic pressure–Diastolic pressure], max arterial pressure, Tau, end-diastolic pressure (EDP) and maximum and minimum dP/dt were determined using LabchartPro software. The aortic augmentation index was identified from the late systolic portion of the arterial pressure wave as described previously [44]. The augmentation index was defined as the height from the augmentation point to the systolic peak of the pressure wave divided by the pulse pressure and is expressed as a percentage.

### Histology

Hearts were fixed in 4% paraformaldehyde and paraffin embedded. 5-μm sections were stained with Masson-trichrome or wheat germ agglutinin (WGA). Masson-trichrome was used to quantify infarct area as previously described [45]. In brief, infarct area was determined using Amira 3D software (Thermo Fisher Scientific, USA) measuring infarcted and non-infarcted myocardium in 10 cross-sections; the right ventricle was excluded from quantification. Percentage infarction was calculated as total pixels in the infarcted left ventricle divided by the total pixel count for the myocardium of the intraventricular septum and left ventricle. Cardiomyocyte size was determined from 8 μm thick transverse cross-sections, cut from paraffin embedded blocks and stained with WGA. Sections were visualised at x20 magnification using the Axioskope plus microscope (Zeiss, Oberkochen Germany). Images were acquired with a DP72 camera (Olympus, Tokyo, Japan). Cell boundaries outlined by WGA were traced and the cardiomyocyte cross-sectional area was measured. Only intact cardiomyocytes where the nuclei could be seen were traced. 50 cardiomyocytes per section from 10 random cross sections throughout the heart were measured. All image analysis was performed using Image J software.

### RNA isolation and quantitative RT-PCR

For RNA isolation heart tissue from the 2-week study was collected, separated into two, the left ventricle and the right ventricle and septum, snap frozen and stored at -80°C until used. Total RNA was extracted using the TRI Reagent solution (Applied Bio-systems, Thermo Fischer Scientific, USA) and transcribed into cDNA using the High Capacity cDNA Reverse Transcription Kit (Applied Bio-systems, Thermo Fischer Scientific, USA). Quantitative RT-qPCR was performed on a ViiA™7 Real-time PCR system (Thermo Fischer Scientific, USA) using QuantiFast EverGreen PCR Kit (QIAGEN). Data were analysed using the comparative ^ΔΔ^CT method [46].

### Statistical analysis

Statistical analysis was generally performed using R version 2.3.2 [47]. We created a combined treatment (Sham or MI), genotype (WT or VDR) and time point (2W or 8W) effect which was then fit as a fixed categorical explanatory variable. The assumptions for linear models (normality and variance homogeneity of residuals) were checked and met for all models. Package lsmeans [48] was then used to calculate least square means. Correction for multiple testing was performed using Bonferroni implemented in package multcompview [49] and p-values < 0.05 were considered as statistically significant. In the experiment with normal and rescue diet, the data were analysed by 3-way ANOVA with the fixed factors genotype (WT or VDR), treatment (Sham or MI), and diet (normal or rescue diet), using IBM SPSS for Windows 24.0 (IBM Corp., Armonk, NY).

## Results

### Survival and infarct area is similar in normocalcaemic VDR mutant and WT mice after MI

**Fig 1:**
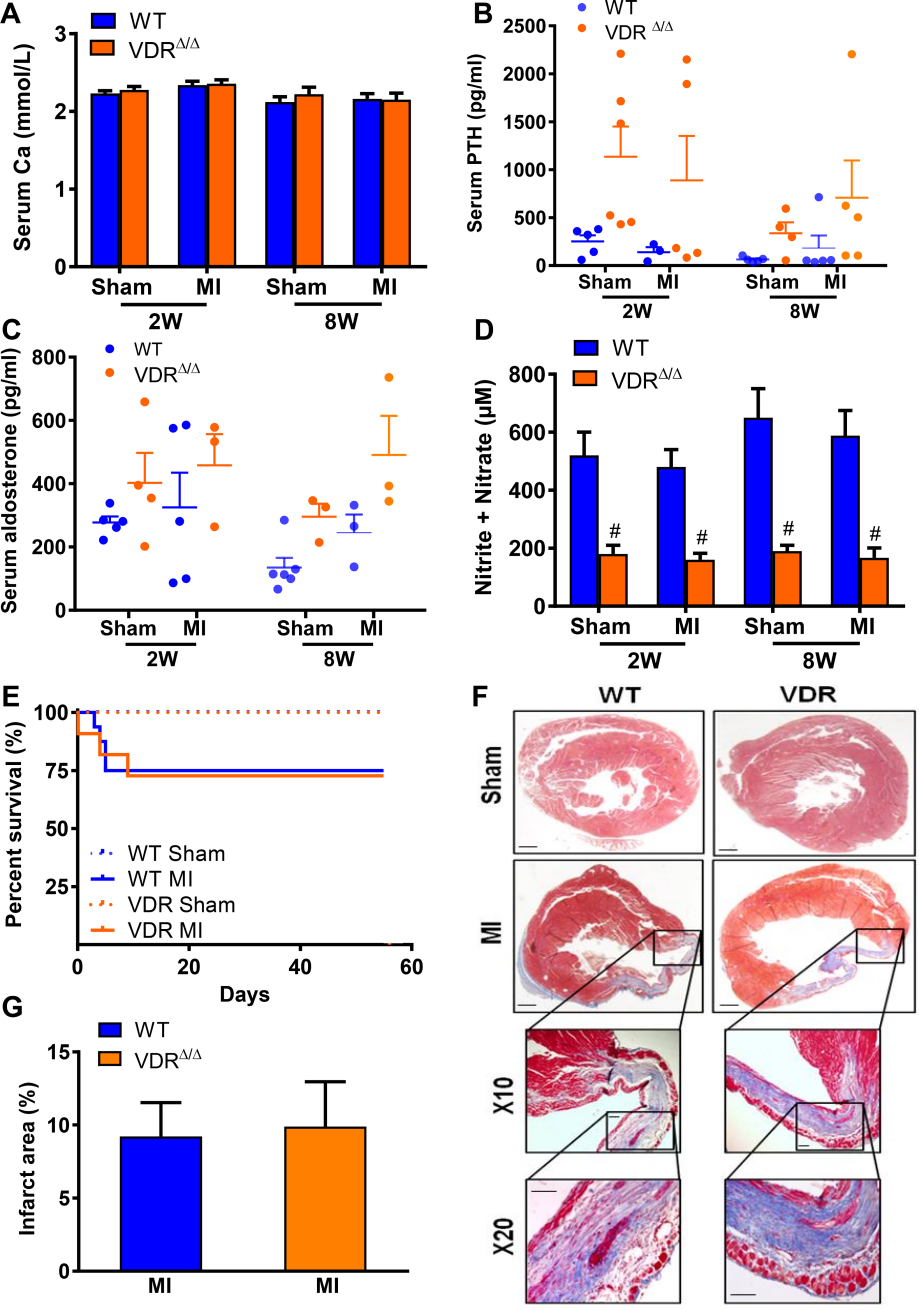
Normocalcaemic mice with a non-functioning VDR show similar survival and infarct area after MI, relative to WT mice. Serum calcium **(A)**, serum PTH **(B)**, aldosterone **(C)**, NO production assessed by measuring nitrite and nitrate levels in urine **(D)** of WT and VDR mutant mice, 2 (2W) and 8 weeks (8W) following sham (S) or MI (M) surgery. Survival curve **(E)**, Masson trichrome staining of heart sections **(F)**, and quantification of infarct area **(G)**, in WT and VDR mutant mice, 8W post-MI. Data represent the mean ± SEM of n = 4–13 animals per group. Individual values are given in S1 Data. * p < 0.05, ** p < 0.01, *** p < 0.001, vs. sham mice of the corresponding genotype; # p < 0.05 vs. WT by least square means linear model with Bonferroni post hoc correction for multiple testing.

Earlier studies examining the effect of VDR loss on MI-induced heart failure progression reported higher mortality and accelerated cardiac pathology in hypocalcaemic VDR mutant mice on a normal diet, 4 weeks post-MI [8]. To assess the isolated effects of lacking vitamin D signalling in the absence of hypocalcemia on survival and heart function after MI, we analysed sham and MI, WT and VDR mutants on rescue diet at 2 and 8 weeks post-surgery. In accordance with our earlier reports [43], the rescue diet largely protected 3-month-old VDR mutants against the development of hypocalcemia and hypophosphatemia (Fig 1A, Table 1). Although serum intact PTH levels tended to be non-significantly elevated in VDR mutant mice (Fig 1B), both sham and MI VDR mutants on rescue diet were normocalcaemic, normophosphatemic, and normonatremic, and showed unchanged serum aldosterone levels compared with WT sham and MI mice (Fig 1A–1C, Table 1). Similar to our previous results demonstrating reduced aortic expression of eNOS and lower bioavailability of NO in VDR mutant mice [19], sham and MI VDR mutants were characterised by diminished urinary nitrite/nitrate excretion, relative to WT mice (Fig 1D). Notably, however, WT and VDR mutant mice showed comparable survival rates and infarct area as shown histologically by Masson trichrome staining (Fig 1E–1G, Table 1). These data indicate that the absence of a functioning VDR is not associated with impaired survival or increased infarct area after MI in normocalcaemic VDR mutants on rescue diet.

**Table 1:** Supporting biochemistry, echocardiography and hemodynamic parameters for WT and VDR animals.

	2-week time point				8-week time point			
Parameter (Unit)	WT		VDR^Δ/Δ^		WT		VDR^Δ/Δ^	
	Sham	MI	Sham	MI	Sham	MI	Sham	MI
***Serum***								
Phosphate (mmol/L)	3.44 ± 0.23	3.25 ± 0.32	3.23 ± 0.28	3.81 ± 0.32	2.95 ± 0.16	2.97 ± 0.16	2.38 ± 0.22	2.61 ± 0.21
Sodium (mmol/L)	147 ± 1.8	143.6 ± 2.7	146.7 ± 2.2	146.8 ± 2.5	131.7 ± 3.1	138.8 ± 3.1	140.7 ± 4.2	141 ± 4
***Urinary***								
Phosphate/Crea (mmol/mmol)	22.7 ± 3.4	15.9 ± 3.8	30.8 ± 2.5	30.1 ± 3.1	13.4 ± 2.7	14.9 ± 4.4	25.7 ± 3.4	27.1 ± 3.8
Sodium/Crea (mmol/mmol)	54.2 ± 5.5	61.9 ± 8.4	140.9 ± 23.8	130.6 ± 26.0	46.7 ± 4.7	56.8 ± 6.8	84 ± 15.4	148.5 ± 41.7
***Echocardiography***								
LVPWd (mm)	1.05 ± 0.08	0.81 ± 0.1	0.99 ± 0.08	0.81 ± 0.11	1.02 ± 0.08	0.92 ± 0.09	0.91 ± 0.08	0.96 ± 0.1
LVAWd (mm)	1.06 ± 0.06	1.04 ± 0.08	1.09 ± 0.06	1.14 ± 0.09	1.01 ± 0.05	0.77 ± 0.08	0.91 ± 0.07	0.85 ± 0.07
***Arterial catheterisation***								
Max Arterial Pressure (mm Hg)	93.5 ± 4.4	88.9 ± 5.2	93.7 ± 3.1	87.4 ± 4.4	104.8 ± 3.4	92.8 ± 3.4	88.1 ± 3.4	83.1 ± 3.6
MAP (mm Hg)	77.3 ± 4.3	73.3 ± 4.9	77.1 ± 3	71.6 ± 4.3	85.7 ± 2.9	77.2 ± 2.9	72.2 ± 2.5	67.5 ± 2.7
APP (mm Hg)	30.7 ± 2.3	30 ± 2.7	32.8 ± 1.7	30.6 ± 2.3	35.9 ± 2.1	30.6 ± 2.1	30.4 ± 1.8	30.2 ± 1.9
Augmentation Index (mm Hg)	21.4 ± 3.2	25.5 ± 4.7	15.1 ± 2.3	20.7 ± 6.3	19.7 ± 4.3	8.5 ± 2.2	11.4 ± 1.6	7.3 ± 1
***Cardiac catheterisation***								
Min dP/dt (mm Hg/s)	-6412 ± 614	-4852 ± 709	-6526 ± 434	-4721 ± 614	-8298 ± 484	-5297 ± 484	-6364 ± 409	-5740 ± 442
Tau (s)	0.015 ± 0.002	0.02 ± 0.002	0.014 ± 0.001	**0.02 ± 0.002** *	0.012 ± 0.001	0.017 ± 0.001	0.013 ± 0.001	0.015 ± 0.001
EDP (mm Hg)	7.9 ± 1.8	8.3 ± 1.7	10.6 ± 1.0	**7.84 ± 1.5** *	11.5 ± 2.5	14.2 ± 2.5	5.05 ± 2.2	10.4 ± 2.3

**Abbreviations:** Wild-type, (WT); Vitamin D receptor, (VDR^Δ/Δ^); myocardial infarction, (MI); Crea, creatinine; left ventricular posterior wall diastole, (LVPWd); left ventricular anterior wall diastole, (LVAWd); mean arterial pressure, (MAP); arterial pulse pressure, (APP); end-diastolic pressure, (EDP); millimeters, (mm); seconds (s).

* p < 0.05 versus sham of corresponding genotype.

### WT and VDR mutant mice on rescue diet do not show differences in cardiac function or hemodynamic parameters post-MI

**Fig 2:**
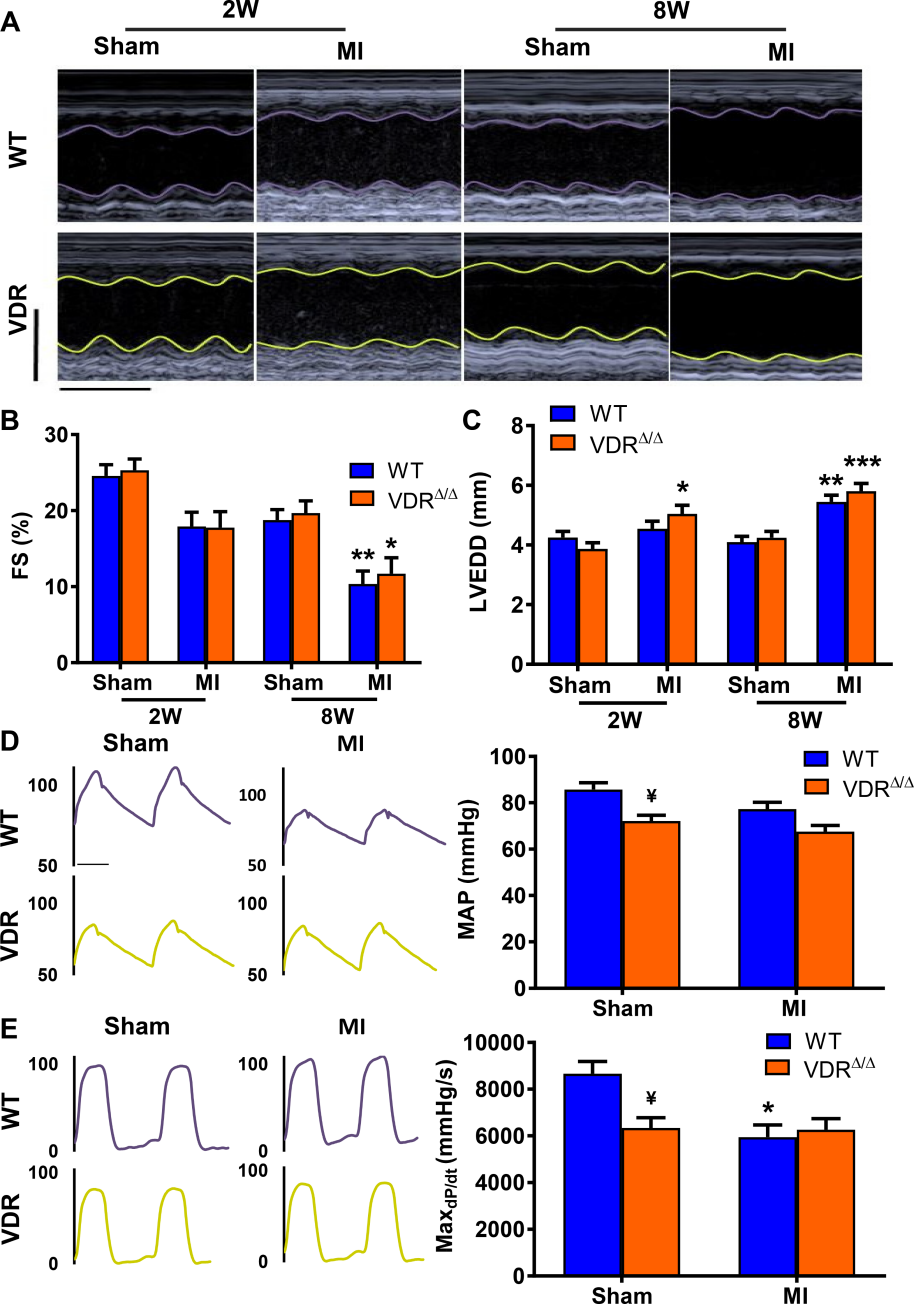
Cardiac function and hemodynamic parameters are equally impaired in WT and in VDR mutants post-MI. Representative M-mode echocardiography images **(A)**, fractional shortening (FS) **(B)**, and left ventricular end diastolic dimension (LVEDD) **(C)** of WT and VDR mutants 2 weeks (2W) and 8 weeks (8W) following sham or MI surgery. Representative aortic blood pressure traces and left ventricular pressure curves, mean arterial pressure (MAP) and rate of left ventricular pressure rise in early systole (Max_dP/dt_) **(D+E)**, of WT and VDR mutants 8W post sham or MI. Groups sizes n = 4–8 per group. Individual values are given in S1 Data. * p < 0.05; ** p < 0.01, *** p < 0.001 versus sham of corresponding genotype; ¥ p < 0.1 vs. WT sham by least square means linear model with Bonferroni post hoc correction for multiple testing. Scale bars in A; X-axis 0.1 seconds, Y-axis 5 mm.

We next asked the question whether the MI-induced functional impairment and heart failure progression differs between normocalcaemic VDR mutants on rescue diet and WT mice. Analysis of functional echocardiographic parameters revealed that induction of MI led to a significant reduction in fractional shortening and an increase in left ventricular end diastolic dimension by 8 weeks post-MI (Fig 2A–2C). However, there were no differences between the genotypes. Aortic catheterisation revealed unchanged mean arterial pressure after MI in both VDR-ablated and WT animals at the 2- and 8-week time points (data not shown, Fig 2D). Cardiac catheterisation revealed a reduction in contractility as evidenced by maxdP/dt in WT mice, 8 weeks post-MI. Sham VDR mutants at the 8-week time point showed a tendency towards a reduction in maxdP/dt, relative to WT mice (Fig 2E). However, there was no further impairment of cardiac contractility in VDR mutants after MI, so that MI WT and MI VDR mutants did not differ in cardiac contractility, 8 weeks post-MI (Fig 2E). Collectively, these data do not support the notion that lack of VDR signalling per se aggravates cardiac dysfunction post-MI.

**Fig 3:**
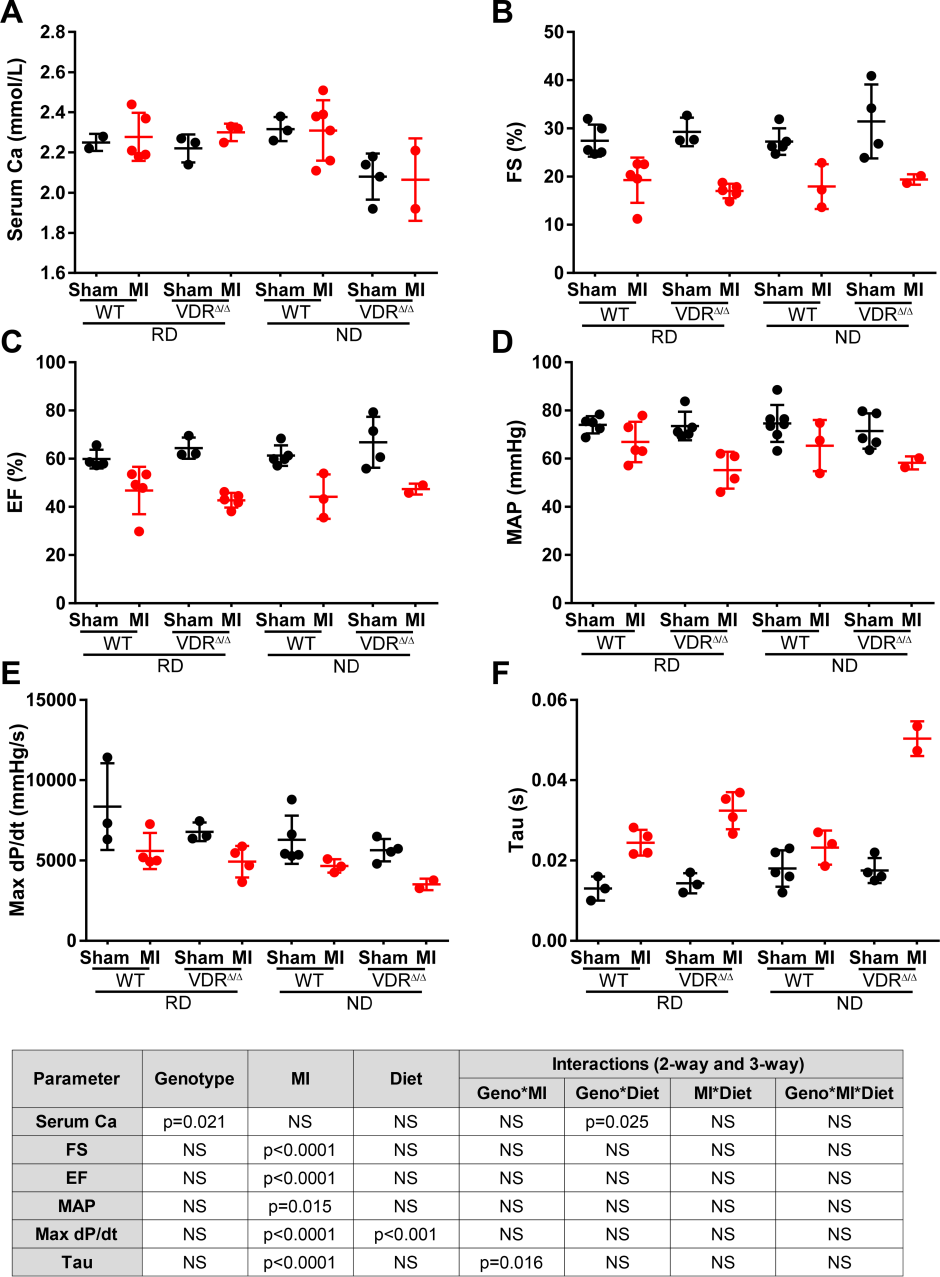
Cardiac function after MI is similar in VDR mutant mice on normal and rescue diet. Serum calcium **(A),** fractional shortening (FS) **(B)**, ejection fraction (EF) **(C)**, mean arterial pressure (MAP) **(D)**, rate of left ventricular pressure rise in early systole (MaxdP/dt) **(E)**, and left ventricular relaxation time constant (Tau) **(F)** in WT and VDR mutant mice on normal diet (ND) or rescue diet (RD), 4 weeks after sham or MI surgery. Groups sizes n = 2–7 per group. Individual values are given in S1 Data. Inset shows results of 3-way ANOVA.

To further explore the interaction between VDR signalling and hypocalcaemia for heart function post-MI, we performed an additional experiment with WT and VDR deficient mice on normal and rescue diet. As expected, Sham and MI VDR deficient mice on normal diet were hypocalcaemic (Fig 3A). MI resulted in left ventricular systolic and diastolic dysfunction as evidenced by lower fractional shortening (Fig 3B) and ejection fraction (Fig 3C), hypotension (Fig 3D), reduced contractility (maxdP/dt, Fig 3E), and increased relaxation time constant (Tau, Fig 3F), relative to Sham mice. However, with the exception of left ventricular contractility (Fig 3E), genotype and diet had no significant influence on heart function post-MI. Interestingly, contractility was slightly but significantly higher in mice on rescue diet, independent of genotype (Fig 3E). In addition, we found an interaction between genotype and MI for the left ventricular relaxation time constant (Fig 3F), i.e., the normal diet and subsequent hypocalcaemia aggravated diastolic dysfunction only in VDR mutant but not in WT mice. The latter finding may point to a modulating influence of hypocalcaemia and sHPT on left ventricular diastolic function in VDR deficient mice post-MI.

### The development of heart hypertrophy and the pro-inflammatory response after MI is comparable in VDR mutant and WT mice on rescue diet

**Fig 4:**
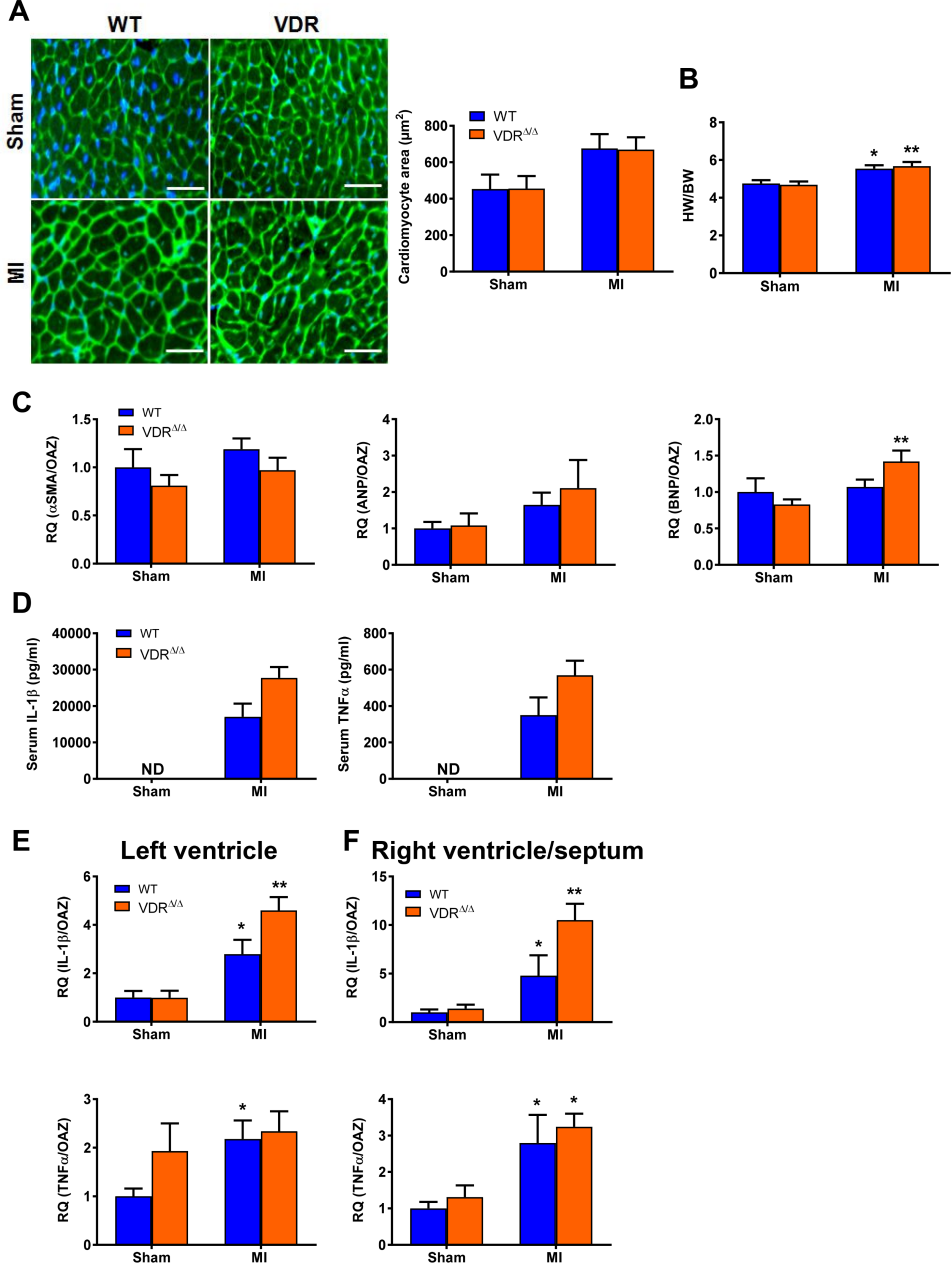
Development of heart hypertrophy and pro-inflammatory response post-MI do not differ between VDR-ablated mice and WT mice on rescue diet. Example of histological sections from the left ventricle, stained with wheat germ agglutinin (WGA) and accompanying quantification of cardiomyocyte area **(A),** heart/body weight (HW/BW) ratio **(B),** left ventricle *αSMA* (α-smooth muscle actin), *ANP* (atrial natriuretic peptide) and *BNP* (B-type natriuretic peptide) mRNA expression **(C)**, serum IL-1β and TNF-α levels **(D),** and mRNA expression of *IL-1β* and *TNF-α* from the left ventricle and from the right ventricle and septum **(E+F)** of WT and VDR mutants at 8 weeks (8W) post sham (S) or MI (M) surgery. Groups sizes for A, n = 4–6 and for B and C, n = 5–7. Individual values are given in S1 Data. * p < 0.05; ** p < 0.01 versus sham of corresponding genotype by least square means (LSM) linear model with Bonferroni post hoc correction for multiple testing. not detectable (ND).

Vitamin D has been implicated in anti-hypertrophic signalling in the heart [50]. Therefore, we next tested the effects of MI on hypertrophy and pro-inflammatory parameters in the heart and in the blood of WT and VDR mutant animals on rescue diet. In accordance with our earlier data in 3-month-old VDR mutant mice [19], there were no differences in cardiomyocyte area as measured by wheat germ agglutinin histology or in heart/body weight ratio between sham WT and VDR mutant mice (Fig 4A and 4B). Moreover, a similar MI-induced increase in cardiomyocyte area and heart/body weight ratio was observed in VDR mutant and WT mice, 8 weeks post-MI (Fig 4A and 4B). Left ventricular mRNA expression of typical markers of cardiac hypertrophy such as α-smooth muscle actin (*α-SMA*), atrial natriuretic and B-type natriuretic peptides (*ANP* and *BNP*) tended to be higher after MI (statistically significant only for *BNP*), but again no difference between WT and VDR mutant mice. MI was associated with profound increases in the serum concentrations of IL-1ß and TNF-α in WT and VDR mutant mice, 8 weeks after MI (Fig 4D). Similarly, mRNA expression of *IL-1β* and *TNF-α* in the left ventricle and in right ventricle and septum was increased in MI mice, 2 weeks after MI (Fig 4E and 4F). However, VDR mutants showed comparable *IL-1β* and *TNF-α* mRNA expression levels in both the left ventricle and the right ventricle/septum compared to WT mice post-MI (Fig 4E and 4F). Taken together, these findings suggest that the absence of vitamin D signalling does not change the development of cardiac hypertrophy after MI, and that the MI-induced upregulation of local and systemic pro-inflammatory signals occur in a vitamin D-independent manner.

## Discussion

Vitamin D plays a critical role in mineral homeostasis, bone biology, immunity [51–54] and has also been implicated in the pathophysiology of CVD. However, this still remains a controversial issue, because a recent, well-powered Mendelian randomisation study did not support an association between 25OHD blood levels and CVD [7]. The latter study suggests that the previously reported associations between circulating 25OHD levels and CVD were possibly confounded. On the other hand, there is strong experimental evidence from global and cardiac-specific VDR-ablated mice that the vitamin D hormone plays an important role in the physiological regulation of LV function and vascular tone [19;50]. In the current study, we focused on the functional role of vitamin D signalling for cardiac function after experimental MI. We found that normocalcaemic mice with a non-functioning VDR maintained on rescue diet were characterised by similar deteriorations in post-ischemic cardiac function and similar increases in mortality and pro-inflammatory signalling to those observed in WT animals. Therefore, lack of vitamin D signalling per se does not aggravate cardiac pathology after MI.

Our study is in disagreement with an earlier study reporting that global VDR knockout mice show decreased survival and impaired cardiac function post-MI, relative to WT controls [8]. However, the latter study was performed on a normal rodent diet which is known to induce severe sHPT in VDR-ablated mice due to an intestinal calcium absorption defect and subsequent hypocalcaemia [43]. Therefore, it is not possible to dissect the effects that direct loss of vitamin D signalling has from those of elevated PTH and hypocalcaemia on cardiac function in global VDR knockout mice kept on a normal diet. Bae and coworkers [8] did not report blood calcium levels in their VDR knockout mice on normal diet. In our experimental setting, moderate hypocalcaemia (Σ2.1 mmol/L total calcium) in VDR mutant mice on normal diet did not have major detrimental effects on heart function after MI relative to normocalcaemic VDR mutant mice on rescue diet. We do not have a conclusive answer for the discrepancies between our study and that of Bae et al [8]. However, a possible explanation may be that only more severe hypocalcaemia may impair cardiac function after MI in VDR deficient mice. In any case, our findings suggest that sHPT and hypocalcaemia, rather than the absence of cardiac VDR, may be primarily involved in the decline of cardiac function post-MI in global VDR deficient mice on a normal diet reported earlier [8]. Similarly, clinical studies have linked sHPT to elevations in blood pressure and cardiac hypertrophy [55]. Furthermore, partial correction of sHPT following treatment with vitamin D analogues has been shown to improve cardiac function [56].

Animal models with tissue-specific VDR deletion circumvent the problem of pleiotropic effects of vitamin D signalling, and the endocrinological disorders linked to vitamin D deficiency. Using this approach, VDR-expressing cardiomyocytes as well as vascular endothelial cells were proposed as a cellular target for protective vitamin D actions in the cardiovascular system [20]. Cardiac-specific VDR deletion was reported to directly cause cardiomyocyte hypertrophy [24]. However, in the current study, we did not observe cardiomyocyte hypertrophy in sham VDR mutant mice. Furthermore, MI-induced cardiac hypertrophy was not greater in VDR mutant mice compared to WT animals, speaking against a major role of deficient VDR signalling in the development of MI-induced cardiac hypertrophy. A better understanding of the role of the VDR in MI-induced pathology may come in the future from experimental MI models in cardiomyocyte-specific conditional VDR knockouts. However, these experiments have not been performed thus far.

The focus of the present study was to examine the effects of defective VDR signalling in normocalcaemic global VDR mutant mice on cardiovascular outcomes following experimental MI. It is known that 9-month-old global VDR mutants on rescue diet develop arterial stiffening, increased pulse pressure, and increased aortic collagen content due to a long-term reduction in endothelial NO production [19]. To avoid arterial stiffening and increased afterload as possible confounders, we performed our experiments in 3-month-old VDR mutants, in which arterial stiffening is still absent [19]. In line with our earlier report [19], we found distinctly reduced urinary total nitrite/nitrate excretion in VDR mutants in this experiment, relative to WT mice, indirectly suggesting lower endothelial NO production. It is conceivable that reduced NO production and endothelial dysfunction might additionally impair cardiac function after MI in VDR mutants. Endothelial dysfunction and increased arterial stiffness are common events associated with MI and the progression of ischemic and chronic heart failure in the aged population [21]. However, we did not observe such an effect. In addition, MI neither modulated total urinary nitrite/nitrate excretion nor significantly increased augmentation index in WT or VDR mutant mice.

Inflammation plays an essential role in scar formation and cardiac remodelling post-MI. There is good evidence for a role of vitamin D signalling in inflammatory and fibrotic processes [57–61]. Indeed, an earlier study in VDR knockout mice kept on a normal diet showed accelerated cardiac inflammation and fibrosis compared with WT mice after MI [8]. Secreted IL-1β and TNFα have a critical role in the post-ischemic remodelling by stimulating inflammatory cell accumulation, inflammatory cytokine production, myofibroblast differentiation, extracellular matrix degradation and collagen production [62–66]. Clinical and animal studies have suggested that vitamin D deficiency may be associated with increased circulating IL-1β and TNFα as well as with increased fibroblast proliferative activity [67–69]. Here, we found that lack of vitamin D signalling in normocalcaemic VDR mutants led to unchanged circulating IL-1β and TNFα levels, and to a similar upregulation of cardiac mRNA expression of these pro-inflammatory cytokines, 2 weeks post-MI. Of note, we found little evidence of cardiac interstitial fibrosis in WT or VDR mutant mice, 8 weeks post-MI.

In conclusion, our study suggests that lack of vitamin D signalling in normocalcaemic global VDR mutants on rescue diet does not negatively influence cardiac function or the development of hypertrophy after experimental MI. Although not supported by our data, it was previously reported that global VDR knockouts on normal diet show profoundly impaired heart function and survival after MI [8]. Therefore, our findings indirectly suggest that sHPT and hypocalcaemia may negatively impact cardiac function and remodelling following MI, and hence promote the development of heart failure. Thus, our study supports the notion that not the lack of vitamin D signalling *per se*, but rather the often overlooked consequences of sHPT, induced by vitamin D deficiency, have detrimental effects on cardiac function post-MI, a concept which may have important clinical implications. Future studies are needed to address the mechanisms of how elevated PTH levels in vitamin D deficiency contribute to the progression of cardiovascular pathology. Improved insight into these mechanisms may lead to more efficient treatment strategies in MI patients.

## Supporting information

S1 Data Data used for making graphs for “Lack of vitamin D signalling per se does not aggravate cardiac functional impairment induced by myocardial infarction in mice”. (XLSX)
